# Dendritic Cells Transduced with Single Immunoglobulin IL-1-Related Receptor Exhibit Immature Properties and Prolong Islet Allograft Survival

**DOI:** 10.3389/fimmu.2017.01671

**Published:** 2017-11-30

**Authors:** Zhicheng Xue, Xuzhi Zhang, Maogen Chen, Xinjun Lu, Ronghai Deng, Yi Ma

**Affiliations:** ^1^Department of Organ Transplantation, First Affiliated Hospital of Sun Yat-sen University, Guangzhou, China

**Keywords:** single immunoglobulin IL-1-related, TIR8, dendritic cell, immune tolerance, islet transplantation, adenovirus transduction

## Abstract

Members of toll-like receptor-interleukin 1 receptor signaling [TLR/IL-1R (TIR)] superfamily mediate maturation of dendritic cells (DCs) and launch immune response in transplanted organs. In this study, we hypothesized that TIR8, also known as single immunoglobulin IL-1-related receptor (SIGIRR) molecule, refrain DCs from maturation and induce immune tolerance of transplanted organ. DCs were transduced with the recombinant adenovirus Ad5F35 to highly express SIGIRR (DC-SIGIRR), then injected to murine recipient before islet transplantation. It revealed that DCs transduced with SIGIRR had low expression of major histocompatibility and costimulatory molecules along with strong phagocytic ability *in vitro* assay. The data demonstrated that recipients treated with DC-SIGIRR had satisfying islet allograft function and long survival times, with an increase of Treg and reduction of Th17 in both spleen and draining lymph nodes *in vivo*. Therefore, genetic modification of SIGIRR inhibits DC activation and maturation, affects differentiation of T cell subsets, protects allograft biological function, and prolongs graft survival.

## Introduction

With the increasing prevalence of diabetes and surgical advances made in clinical transplantation over the past decade, doctors found islet transplantation a minimally invasive therapeutic treatment that can effectively recuperate normoglycemia and insulin independence in Type 1 diabetes mellitus without the surgical complications associated with vascularized pancreatic allograft (1, 2). Nonetheless, rejection of allografts continues to hamper the success of islet transplantation. Therefore, it is of critical importance to identify a potent and effective immune therapy for patients received islet transplantation.

Dendritic cells (DCs), the potent antigen presenting cells (APCs), play a critical role in activating antigen-specific transplant immune response. DCs provide an initiating signal to induce innate and specific immunity, as well as transplantation immune reaction (3). With high expression of costimulatory molecules and major histocompatibility complex (MHC) on the cell surface, mature DCs are able to induce immune response while immature DCs, lacking of aforementioned molecules, are likely to cause T cell hyporesponsiveness and polarization of T cell subsets (4), thereby inducing antigen-specific tolerance.

Single immunoglobulin IL-1-related receptor (SIGIRR) molecule, a key member of the toll-like receptor-interleukin 1 receptor signaling (TLR/IL-1R) receptor superfamily, suppresses signaling receptor complexes of IL-1 family members associated with helper T cells differentiation and TLR-mediated activation (5). SIGIRR is expressed by NK cells, B cells, monocytes, and immature myeloid DCs, with reduced expression upon maturation (6, 7). Current evidences suggests that in murine model of kidney allograft acceptance constructed by blockade of costimulation, most SIGIRR−/− grafts were rapidly rejected and SIGIRR deficiency is associated with expansion and maturation of renal resident DC precursors (8). In the skin allograft model, MyD88 deletion abrogated the post-transplant increase in the quantity of mature DCs in draining lymph nodes, further suggesting the importance of TLR-dependent signals in DC maturation (9, 10). All the studies above indicated that SIGIRR is indeed involved in the modulation of DC maturation and function, which have the potential therapeutic implication for inducing immune tolerance by manipulating high expression of SIGIRR in DCs before transplantation.

Given the negative effect of SIGIRR in immune response, we tested the hypothesis that overexpression of SIGIRR inhibits DC maturation and function by transducing DCs with the recombinant adenovirus Ad5F35 to highly express SIGIRR, and examined if DC-SIGIRR prolonged islet allograft survival.

## Materials and Methods

### Animals

Specific pathogen-free (SPF) C57BL/6 mice (H-2^b^) (male, 6–8 weeks old) and SPF BALB/c mice (H-2^d^) (male, 8–10 weeks old) were purchased from the Experiment Animal Center of Sun Yat-sen University (Guangzhou, China). All animal experimental procedures were approved by the Institutional Animal Care and Use Committee (IACUC), Sun Yat-sen University. Mice were housed in an SPF-grade facility with proper ambient temperature and relative humidity, and received humane care in strict accordance with the “3R” Principles of IACUC.

### Reagents

Recombinant murine granulocyte-macrophage colony-stimulating factor (GM-CSF) and interleukin-4 (IL-4) were purchased from PeproTech (NJ, USA). Carboxyfluorescein succinimidylester (CFSE), Cell Stimulation Cocktail plus Protein Transport Inhibitor, Foxp3/Transcription Factor Staining Buffer Set, and rat anti-mouse fluorescence-conjugated CD3, CD4, CD8, CD25, Foxp3, IFN-γ, IL-4, IL-17A, CD11c, CD40, CD80, CD86, and MHC-II (I-A/I-E), as well as their corresponding isotype controls, were purchased from eBioscience (San Diego, CA, USA). Microbead-conjugated anti-CD11c and LS sorting columns were purchased from Miltenyi Biotec (Bergisch Gladbach, Germany). Anti-mouse insulin and glucagon monoclonal antibodies (mAbs) were purchased from Cell Signaling Technology (Danvers, MA, USA). The replication-defective recombinant Ad5F35-SIGIRR-green fluorescent protein (GFP) was constructed by Hanbio Biotechnology Co., Ltd. (Shanghai, China). Enzyme-linked immunosorbent assay (ELISA) kits for INF-γ, IL-4, IL-10, and IL-17 were purchased from RayBiotech (Norcross, GA, USA), and the tumor growth factor (TGF)-b kit was from eBioscience. Streptozotocin (STZ), lipopolysaccharide (LPS), tetramethylrhodamine-dextran (TRITC-dextran, 4,000 MW), Histopaque 1077 were purchased from Sigma-Aldrich (St. Louis, CA, USA). Liberase TL, diphenylthiocarbazone (DTZ), acridine orange (AO), propidium iodide (PI), blood glucose meter, and blood glucose test strips were purchased form Roche (Basel, Switzerland).

### Generation of Bone Marrow Derived Dendritic Cells (BMDCs), Adoptive Transduction of SIGIRR, and Phenotype Analysis

Generation of BMDCs was carried out as previously described (11). In brief, the erythrocytes were lysed after the femurs and tibiae of C57BL/6 mice were flushed. Bone marrow cells were then cultured in 6-well plates with complete culture medium (RPMI 1640 supplemented with 10% FBS, 1% penicillin–streptomycin, 0.1% 2-mercaptoethanol, 1% glutamine, and 40 ng/mL recombinant murine IL-4 and GM-CSF) for 6 days to gain immature DCs (imDCs). By immunomagnetic selection of CD11c+ with anti-CD11c-conjugated microbeads, imDCs were then purified to >99%. Purified DCs were sequentially transduced with Ad5F35-GFP-SIGIRR (DC_SIGIRR) or Ad5F35-GFP (Ad-DCs) at a multiplicity of infection (MOI) of 1:100 for 24 h in serum-free RPMI 1640 (12). Mature DCs (mDCs) were generated by stimulating imDCs with LPS (1 µg/mL) for 24 h. The phenotype expression of CD40, CD80, CD86, and MHC-II were detected by flow cytometry for cell maturation. A total of 2 × 10^6^ SIGIRR-DCs and Ad-DCs were transfected by tail vein injection to recipient mice 1 day prior to islet transplantation.

### Phagocytosis Assay

To investigate the phagocytosis ability of DCs, we measured the mannose receptor-mediated endocytosis by the cellular uptake of TRITC-dextran. Approximately 1 × 10^6^ DCs were resuspended in 250 µL of media with 25 µg/mL TRITC-dextran. After incubation with the TRITC-dextran at 37°C for 2 h, DCs were washed three times with 4°C PBS and 1% FBS, then stained for extracellular CD11c and analyzed by FACS. The fraction of TIRTC-positive cells was calculated after CD11c-positive cells were gated.

### Islet Isolation, Activity Evaluation, and Transplantation

Diabetes was induced in recipient mice (C57BL/6) via a single intraperitoneal injection of STZ (200 mg/kg) after fasting for 4–6 h (13). A mouse was defined as a diabetic recipient when results of three consecutive blood glucose tests under the non-fasting state were >16.7 mmol/L (300 mg/dL) after STZ injection (14). Animal groups consisted of a control group, DC group, Ad-DC group, and DC-SIGIRR group, receiving phosphate buffered saline (PBS), DC, Ad-DC, and DC-SIGIRR, respectively. Islets were isolated from BALB/c mice via reverse perfusion of the common bile duct with Liberase TL and purified by density-gradient centrifugation. Before transplantation, islet purity was tested by DTZ staining and activity was evaluated by AO-PI staining under an inverted fluorescence microscope. Approximately 2 × 10^6^ DCs were adoptively transferred to the recipient mouse on the day before surgery. Then 350 islets were transplanted to subcapsular kidney pouch of diabetic recipients (C57BL/6) created by a glass capillary tube probe. To ease the hyperglycemic stress of the newly transplanted islets, each mouse was injected with one shot of insulin (5 U/kg) at the first day after transplantation. Rejection of islet allografts was defined as blood glucose levels >16.7 mmol/L (300 mg/dL) for at least 2 consecutive days.

### Intraperitoneal Glucose Tolerance Test (IPGTT)

On days 10 and 30 after transplantation (about half of allografts in the Ad-DC group rejected at day 10 while all grafts in control and Ad-DC group rejected at day 30, and the islet-bearing kidneys were removed at these time points), a dose of 2 g/kg glucose was injected intraperitoneally to test glucose tolerance of the recipients after 4–6 h of fasting (15). After glucose injection, blood samples were collected from the caudal vein at every 15 min in 2 h and then analyzed for blood glucose levels with a glucometer. Additionally, IPGTT was repeated after rescission of islet-bearing kidney to confirm that the recipient blood glucose response was due to the bioactivity of donor islet allograft, rather than the recovery of recipient STZ-damaged pancreas.

### Flow Cytometric Analysis

Lymphocyte cells were collected from the recipient spleens and peritoneal draining lymph nodes on day 10 and 30 post transplantation, and stained with the indicated fluorescein mAbs and analyzed on a flow cytometer (Beckman Coulter FACS Gallios, USA). Th1, Th2, Th17, Treg, and intracellular cytokine staining analyses were performed following a 4–6 h stimulation with Cell Stimulation Cocktail plus Protein Transport Inhibitor. The cells were fixed and permeabilized with the Foxp3/Transcription Factor Staining Buffer Set, then stained for intracellular cytokines. Data were obtained using Kaluza for Gallios software, and analyzed with Kaluza Analysis software. Th1 was defined as CD3+/CD8−/IFN+, Th2 was defined as CD3+/CD8−/IL-4+, Th17 was defined as CD3+/CD4+/IL-17+, and Treg was defined as CD4+/CD25+/Foxp3+.

### Enzyme-Linked Immunosorbent Assay

Blood samples were collected from the retro-orbital sinus on days 10 and 30 after transplantation. Samples were properly pretreated and stored at −80°C until analysis. Serum levels of IL-4, IL-10, IL-17, and INF-γ were examined according to the manufacturer’s protocols.

### Hematoxylin and Eosin (HE) and Immunofluorescence (IF) Analyses

Islet allografts were obtained on days 10 and 30 after transplantation. Samples were fixed with 10% formalin, and embedded in paraffin, cut into 3–5 µm thick sections, then stained with anti-mouse insulin or glucagon monoclonal antibodies according to the manufacturer protocols.

### Statistical Analyses

Islet graft survival between groups was analyzed with Kaplan–Meier analysis. Statistical significance between groups was determined by ANOVA. All statistical analyses were performed with SPSS 22.0 software. Values of *P* < 0.05 were considered statistically significant. All data were expressed as mean ± SD.

## Results

### Genetically Modified Ad5F35 Increases the Transduction Efficiency of DCs

**Figure 1:**
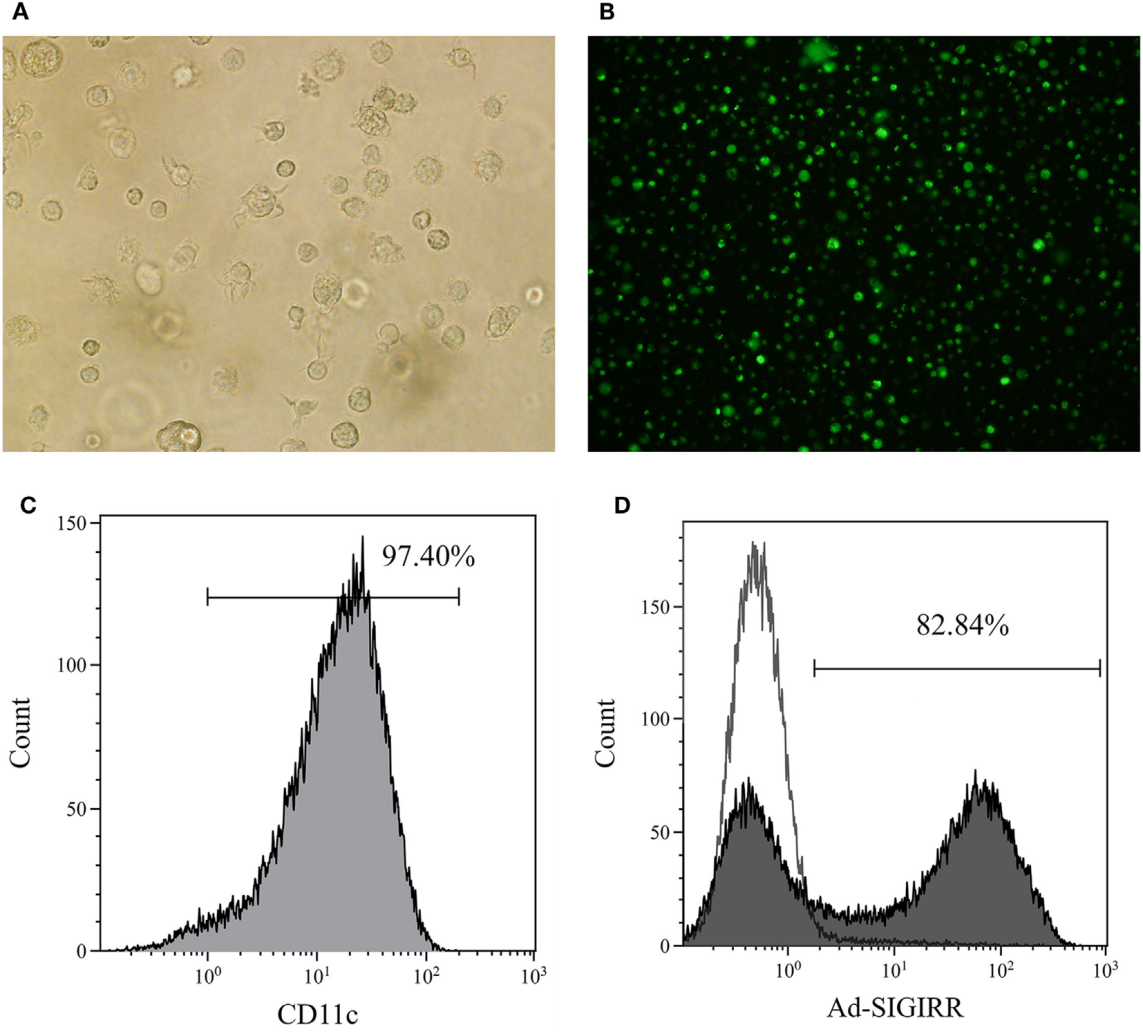
Generation of bone marrow derived dendritic cells (BMDCs) and transduction of single immunoglobulin IL-1-related receptor (SIGIRR). **(A)** Mature DCs (×200); **(B)** the expression of green fluorescent protein in DCs 24 h after Ad5F35-SIGIRR transduction observed in fluorescence microscopy. **(C)** Purity of BMDCs; **(D)** transduction efficiency of Ad-SIGIRR.

The BMDCs were successfully generated with >90% purity after CD11c-positive immunomagnetic selection (Figures 1A–C). The genetically modified recombinant adenovirus Ad5F35 was improved to increase the transduction efficiency (16). Purified DCs were transduced by Ad5F35-SIGIRR-GFP or Ad5F35-GFP at an MOI of 1:100 for 24 h. GFP fluorescence was detected by a fluorescent microscope. Transduction efficiency was analyzed, and approximately 80% of DCs were successfully transduced at day 7 (Figure 1D). To further investigate whether the overexpression can last persistently, DC-SIGIRR was cultured in medium continuously until spontaneous apoptosis (usually 4–6 weeks) and GFP fluorescence could still be observed, indicating that Ad5F35 effectively transferred the SIGIRR gene into DCs persistently.

### DC-SIGIRR Inhibits Expression of Costimulatory Molecules and Remains High Phagocytic Ability in Presence of LPS

**Figure 2:**
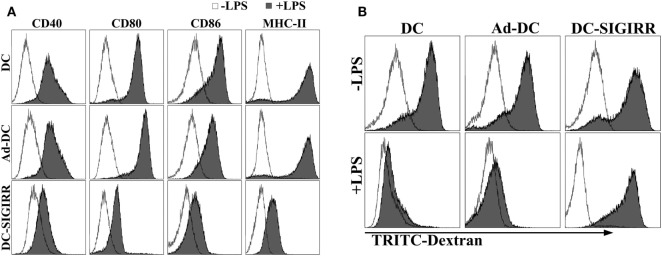
Single immunoglobulin IL-1-related receptor (SIGIRR) molecule decreased the expression of major histocompatibility complex (MHC) and costimulatory molecules. **(A)** DCs transduced with Ad5F35-SIGIRR-green fluorescent protein were stained with fluorescence conjugated MHC-II, CD40, CD80, and CD86 mAbs 24–48 h later. MHC and costimulatory molecules expression were reduced to different degrees compared to normal DCs in presence of LPS; **(B)** DCs remained significant phagocytic ability after LPS stimulation.

Lipopolysaccharide is a representative and well-characterized stimuli of DC maturation (17). To determine the effect of SIGIRR overexpression on DC maturation with the existence of exogenous maturation stimuli, purified iDCs, Ad-DC and DC-SIGIRR were stimulated with LPS (1 µg/mL) for 24 h, then collected and stained with fluorescent-conjugated MHC-II, CD40, CD80, and CD86 mAbs. Following LPS stimulation, DC-SIGIRR group expressed lower levels of CD40, CD80, CD86, and MHC-II than other groups, retaining characteristic of imDCs (Figure 2A). The result indicated that SIGIRR could effectively block the antigen-presenting and T cell activating capacity of DCs by inhibiting expression of costimulatory molecules and MHC. Additionally, there was no significant difference between imDC and Ad-DC group in phenotype expression level before LPS stimulation, indicating that the recombinant adenovirus did not influence DC maturation.

As a prerequisite for antigen-presentation, imDC possesses stronger antigen phagocytosis ability than mDC. To evaluate DC phagocytic ability, TRITC-dextran were incubated with purified iDCs, Ad-DC, or DC-SIGIRR and then assessed by FACS. Both iDCs and DC-SIGIRR exhibited strong phagocytic ability before LPS stimulation (Figure 2B). However, it attenuated the phagocytic ability of iDCs and Ad-DC after maturation induced by LPS. On the other hand, DC-SIGIRR maintained valid phagocytic ability after LPS stimulation.

Given the results of flow cytometry and phagocytosis assay, it is suggested that even in the presence of maturation and activation stimuli, the overexpression of SIGIRR arrested maturation and activation of DCs.

### DC-SIGIRR Protects Allograft Function and Prolongs the Survival

**Figure 3:**
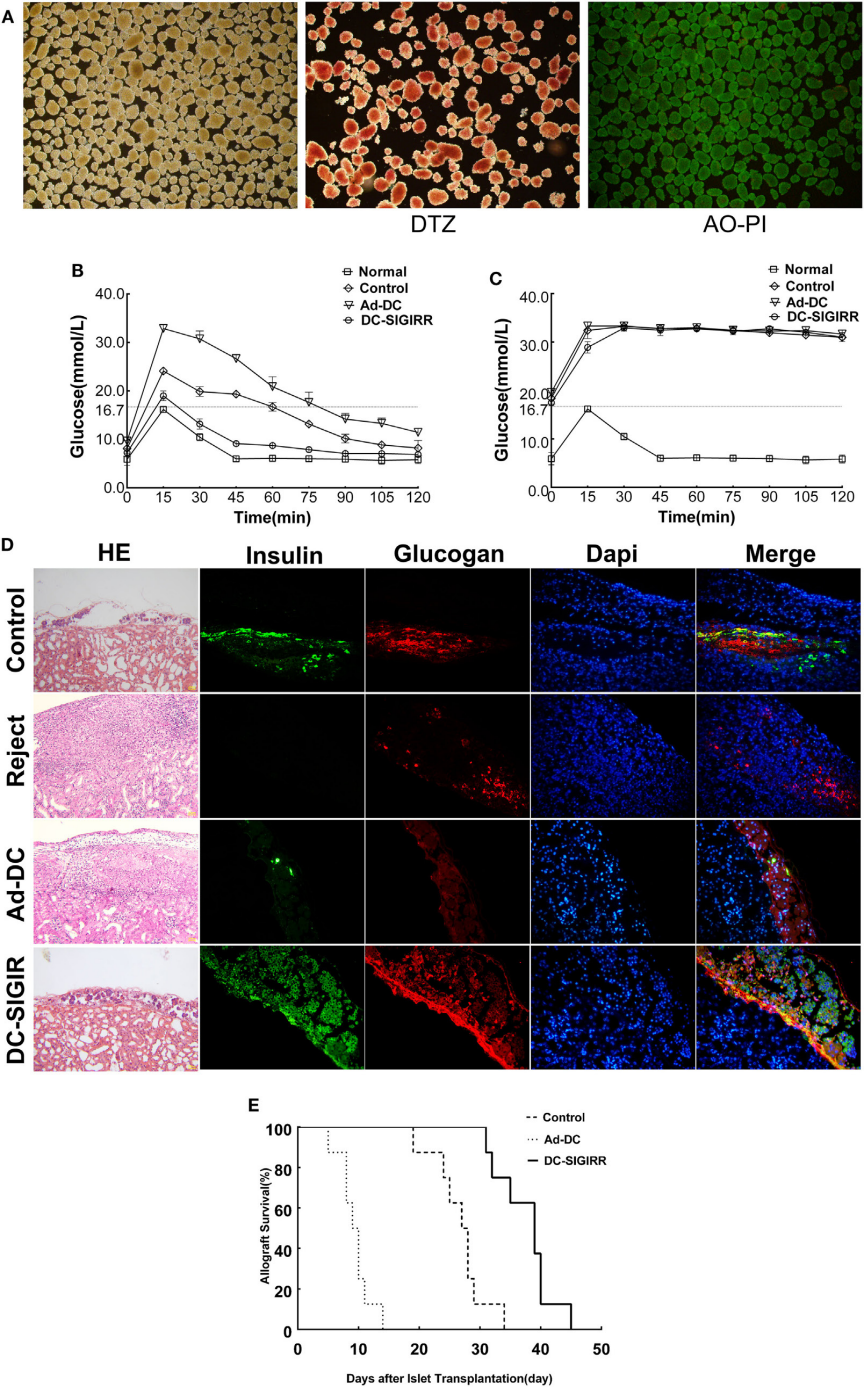
DCs transduced with single immunoglobulin IL-1-related receptor (SIGIRR) molecule prolongs the allografts survival. **(A)** Purified islets isolated from the perfusion with satisfying activity (AO-PI, ×40) and function (DTZ, ×40). **(B)** Intraperitoneal glucose tolerance tests (IPGTTs) were performed on day 10 post transplantation to test recipient glucose tolerance after 4–6 h of fasting. **(C)** IPGTTs were repeated after the removal of islet-bearing kidney to confirm recipient blood glucose response was islet allograft dependent. (The upper limit of the blood glucose meter is 33.3 mmol/L, and if the blood glucose level was above the limit it was recorded as 33.3 mmol/L. Data are expressed as mean only.) **(D)** On day 10 after transplantation, kidneys were removed and stained with the indicated markers. DC-SIGIRR treated recipients displayed higher expression of insulin and glucagon compared to the other groups, indicating greater function and survival of islet grafts (HE × 200, IF × 400). **(E)** Survival of the islet grafts was monitored by daily non-fasting blood glucose levels. Graft survival between groups of PBS control, Ad-DC, and DC-SIGIRR was compared with Kaplan–Meier analysis (data of DC treated group were not shown as results of DC and Ad-DC group are practically the same, *n* = 8). Histological results are shown from a representative recipient from each group.

An islet allograft transplantation model was used to determine the influence of DC-SIGIRR on transplantation immunity. Before transplantation, islet purity was evaluated *in vitro* by DTZ staining and the activity was evaluated by AO-PI staining after isolation. The results revealed a high level of islet purity and activity. Excellent quality islets render the diabetic recipient normoglycemia (<16.7 mmol/L) after transplantation (Figure 3A). To investigate the functional activity of transplanted islet allografts *in vivo*, the IPGTT (2 g/kg) was performed on postoperative day 10 to assess glucose tolerance after 4–6 h of fasting (Figure 3B). All murine recipients exhibited similar baseline of blood glucose levels before IPGITT. Among groups, blood glucose levels elevated steeply after glucose injection, peaked at 15 min, and then descended over time. There was a better and swifter response to the glucose boost in DC-SIGIRR group than other groups. IPGTT was performed once again after the removal of the islet transplanted kidney on day 10 after transplantation and the result verified that the recipient blood glucose response was islet allograft dependent (Figure 3C). The removed kidney was stained with HE/IF for insulin and glucagon to confirm the presence of functional islet grafts (Figure 3D). Survival of the islet grafts were monitored by non-fasting blood glucose levels daily. DC-SIGIRR substantially prolonged the survival time of islet allografts, with a median survival time (MST) of 39 ± 1.27 days compared to 27 ± 0.36 days (*P* < 0.01) in the no treatment control group (Figure 3E). Notably, the survival time of Ad-DC group is astonishingly shorter than the control group. As indicated by the result, we considered the possibility that excess quantity of homologous DCs could exaggerate rejection.

### DC-SIGIRR Induces Expansion of Treg and Secretion of Immunosuppressive Cytokines

**Figure 4:**
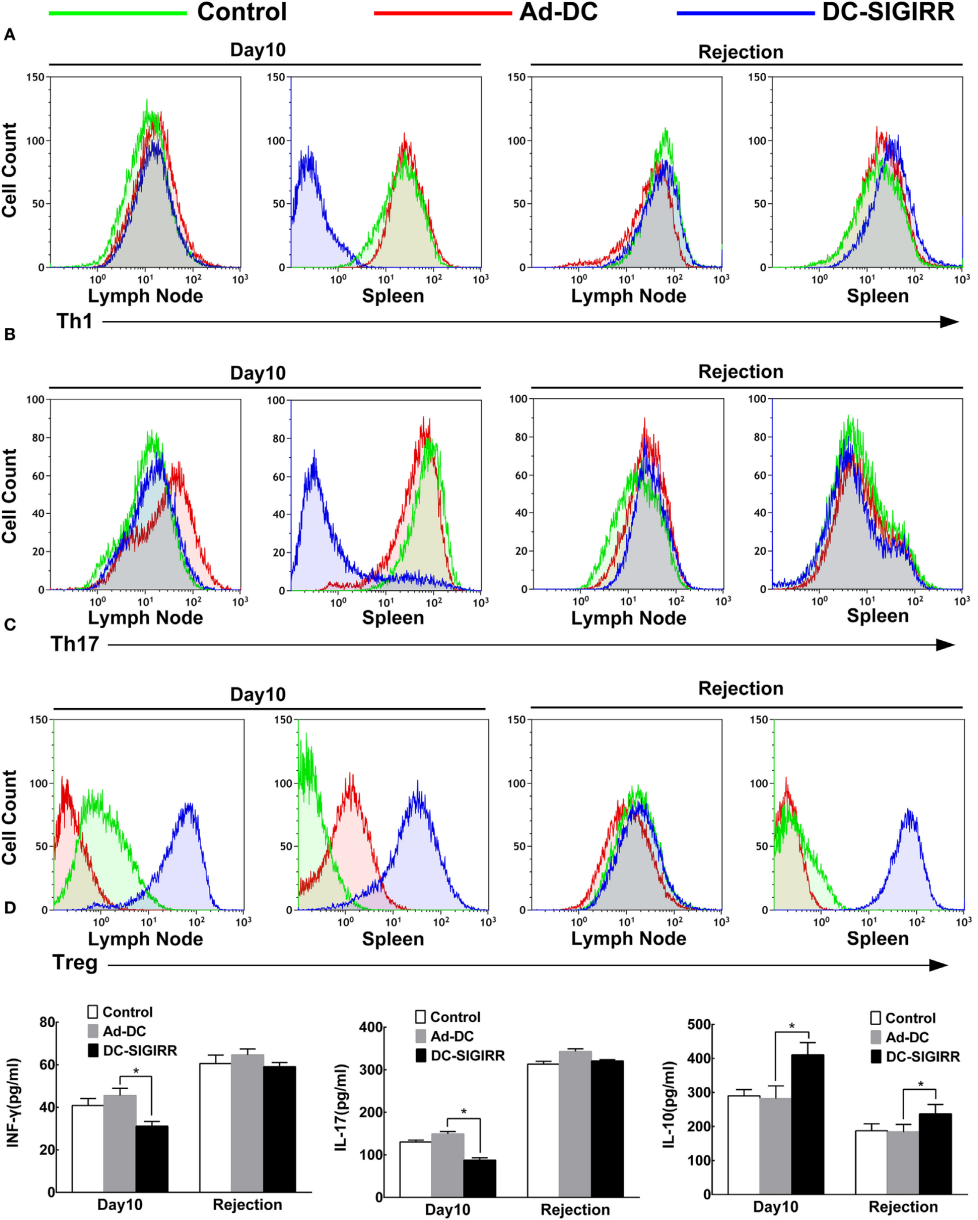
DC-single immunoglobulin IL-1-related receptor (SIGIRR) molecule reduced the populations of Th1 and Th17 cells and their secreted cytokines while increased the IL-10 level and induced expansion of Treg in transplant recipients. **(A–C)** Representative flow cytometry plots showing quantity of Th1, Th17, and Treg lymphocytes in draining lymph nodes and spleens of PBS control, Ad-DC, and DC-SIGIRR on day 10 after transplantation and on the day of rejection. **(D)** Cytokines level of serum were analyzed on day 10 after transplantation and on the day of rejection. Data represent the mean ± SD of three independent experiments. *P < 0.05, *n* = 5.

To determine the underlying mechanisms of DC-SIGIRR treatment to prolong the islet allograft survival in treated mice, we evaluated changes in T cell subsets and their secreted cytokines. Lymphocytes were collected from recipient spleens and draining lymph nodes, then stained with the indicated fluorescein mAbs and analyzed on a flow cytometer. The results revealed that DC-SIGIRR group significantly reduced the population of Th1 and Th17 subsets in splenocytes (*P* < 0.05) compared to the control group and Ad-DC group. Expansion of Treg was observed in both draining lymph nodes and splenocytes in DC-SIGIRR group (*P* < 0.05) (Figures 4A–C). Nevertheless, there were no significant differences in Th2 cell population in neither the lymph nodes nor splenocytes between groups (data not shown).

Serum levels of IL-4, IL-10, IL-17, and INF-γ were also measured (Figure 4D). Compared to the Ad-DC group, INF-γ and IL-17 were found lesser (*P* < 0.01) while IL-10 was significantly increased in the DC-SIGIRR group compared to the other groups. Additionally, there were no significant differences in IL-4 secretion in neither the lymph nodes nor splenocytes between groups (data not shown).

## Discussion

Our present study shows that genetically modified DC-SIGIRR expressed low level of costimulatory molecules, retained strong phagocytic ability, induced expansion of Treg, and prolonged islet allograft survival. SIGIRR, an IL-1R/TLR family member, arrested signaling receptor complexes of IL-1family members associated with T cell subset differentiation and also dampens TLR-mediated activation. The negative regulation on signaling pathway of ILR family and TLRs makes SIGIRR an initial regulator of inflammation, innate immunity, and polarization of T cell orchestrated adaptive responses.

Toll-like receptors initiate signaling pathways that evoke activation of innate immunity, which induces DC maturation and sequentially initiation of adaptive immune responses that provoke allograft rejection. Moreover, mounting evidence indicated that SIGIRR protein indeed participate in regulating the maturation and functional states of DCs. In the study conducted by Noris et al. (8), marked increases both in numbers of intragraft CD11b+ cells and DC and in the fraction of these cells that expressed MHCII were found in *sigirr*−/− kidney grafts compared with *sigirr*+/+. The pathogenetic mechanism underlying the susceptibility of *sigirr*-deficientlpr/lpr mice to autoimmunity diseases is based on enhanced activation of DCs to complexed lupus autoantigens and production of proinflammatory cytokines (18). To obtain more comprehensive information on the effect of SIGIRR, it inspired us to explore the consequences of SIGIRR overexpression in DCs. Thus we increased SIGIRR expression of DCs by genetically modified Ad5F35 virus transduction. Our results revealed that SIGIRR overexpression could steadily refrain DCs from maturation with manifestation of low levels of MHC and costimulatory molecules expression as well as stable phagocytosis activity. It is reported that LPS stimulation leads to considerable decrease of SIGIRR expression in different murine tissues (19), and SIGIRR-deficient mice are more susceptible to the systemic toxicity of LPS (7, 19). However, our results revealed that even with the presence of LPS, SIGIRR could be persistently up-regulated in DC transduced with SIGIRR.

According to the result of preliminary experiment, approximately half of recipients in the Ad-DC group rejected on day 10 while all in the Ad-DC and control group rejected on the day 30 after transplantation. Therefore, we selected day 10 and 30 as a time point to examine the biological differences among groups as well as changes in physiological function of grafts over time before rejection, in addition to the mere comparison of graft survival time. As expected, DC-SIGIRR significantly prolonged the survival time of islet allografts whereas Ad-DC exaggerated allograft rejection. It has been clearly demonstrated by Faustman et al. (20) that islets pretreated with anti-DC antibody prior to transplantation survived in their histoincompatible recipients for >200 days while stable islet allografts rapidly rejected once transplant recipients were injected with DCs 60 days after transplantation, which is consistent with our finding that DCs exaggerate graft rejection. Additionally, many scholars adopted DC modification as a potential strategy to induce transplantation tolerance (21, 22). The aforementioned results demonstrate an important *in vivo* role for DCs in the stimulation of allograft rejection.

Nevertheless, mature DCs may either activate or block Treg generation upon Ag recognition while DCs do not activate T cells instead of directing their differentiation to Treg (23). DCs induce T cell expansion and Th cell subset polarization via MHC and costimulatory molecules (24). And SIGIRR had negative effect on DC maturation. However, upon stimulation of transplant immunity, DCs mature and produce high amounts of detrimental cytokines, like INF-γ and IL17 to allograft, which inhibits generation and function of Treg and tips the balance of T cell subsets toward Teff (25). It was reported that SIGIRR deletion severely impaired the appearance of Treg in graft while the accumulation of Treg in accepted grafts was protective in various experimental models of transplant tolerance by suppressing Teff function (26). We then reasoned that persistent overexpression of SIGIRR in DC could increase the expansion of Treg, secretion of immunosuppressive cytokines and consequently improve graft survival. Consistently, we found that DC-SIGIRR-treated recipients had better glucose tolerance than the other groups, and flow cytometry analysis demonstrated an expansion of Treg and reduction of Th1 and Th17. This change in T cell subset helps to alleviate allograft rejection (27), thereby prolonging the survival of the islet allografts in our model. The reduction of INF-γ and IL-17 serum levels further confirmed this result. As a well-known immunosuppressive cytokine, IL-10 is a key mediator of immune tolerance (28). We observed higher expression of IL-10 in allograft recipients, demonstrating that IL-10 is of great importance for inducing transplant acceptance and tolerance (29).

Though SIGIRR significantly prolongs the islet allograft survival time, transplantation rejection occurred eventually. Given the limitation of this study, we infer that the rejection could be resulted from the following aspects. First, DC-SIGIRR could not self-renewal like progenitor cells, which means that all of DC-SIGIRR doomed to apoptosis in the end and consequently caused time-limited overexpression of SIGIRR in recipients. On the other hand, proportion of DC-SIGIRR decreased over time concurrently with continuous generation of protogenetic DCs in recipients, which gradually exaggerates rejection. Finally, it is a consensus that the molecular mechanism of transplantation tolerance involves complex regulatory network of immune system, instead of single certain pathway (30, 31). Based on this notion, it is reasonable to believe that even persistently overexpression of SIGIRR is not enough to induce stable tolerance. Therefore, our study demonstrates the therapeutic potential of SIGIRR overexpression in DCs in tolerance induction, which united other molecular immune therapies to achieve graft tolerance.

Taken together, injection of DC-SIGIRR prior to transplant prolonged allograft survival, but all of the islet allografts were eventually rejected. This leads us to wonder if persistent generation of DC-SIGIRR in recipients may eventually induce immune tolerance. Constitutive expression and combination therapeutic treatment of SIGIRR need to be further explored. Moreover, *sigirr* deficiency specifically in DCs is needed for further experiments in order to fully explore the mechanism of SIGIRR in modulating DCs function in mouse islet transplant model.

## Conclusion

Single immunoglobulin IL-1 receptor-related genetic modification efficiently confers low expression of MHC and costimulatory molecules on DCs, which could induce expansion of Treg and prolonged islet allograft survival in mice. These findings may have important therapeutic implications for new immunomodulating protocols, such as manipulating SIGIRR expression in APCs before transplantation.

## Ethics Statement

This study was carried out in accordance with the recommendations of Experimental Animals Welfare Guideline of the Sun Yat-sen University Animal Care and Research Committee. The protocol was approved by the Sun Yat-sen University Animal Care and Research Committee.

## Author Contributions

ZX and XZ contribute equally to this work and considered as co-first authors. ZX analyzed the data, interpreted the results, and drafted the manuscript of the work; ZX, XL and XZ constructed the animal models; ZX, XZ, RD and MC analyzed the data; ZX, XZ, XL, MC and YM interpreted the results; YM and RD designed the work and revised it critically.

## Conflict of Interest Statement

The authors declare that the research was conducted in the absence of any commercial or financial relationships that could be construed as a potential conflict of interest.
